# CD14^+^HLA-DR^low/−^ expression: A novel prognostic factor in chronic lymphocytic leukemia

**DOI:** 10.3892/ol.2014.2808

**Published:** 2014-12-17

**Authors:** JINLIN LIU, YONGLIE ZHOU, QIANG HUANG, LIANNV QIU

**Affiliations:** 1 Department of Clinical Laboratory, Zhejiang Provincial People’s Hospital, Hangzhou, Zhejiang 310004, P.R. China; 2 Department of Hematology, Zhejiang Provincial People’s Hospital, Hangzhou, Zhejiang 310004, P.R. China

**Keywords:** chronic lymphocytic leukemia, CD14^+^HLA-DR^low/−^, prognostic factor

## Abstract

Currently, no prognostic factors exist for determining the host immune status of chronic lymphocytic leukemia (CLL) patients. Therefore, the present report analyzed cluster of differentiation 14 (CD14)^+^ human leukocyte antigen (HLA)-DR^low/−^ myeloid-derived suppressor cells (MDSC) from 49 CLL patients and demonstrated that these cells were significantly expanded in all CLL patients when compared with monoclonal B cell lymphocytosis patients and healthy volunteers. Furthermore, upregulation of CD14^+^HLA-DR^low/−^ MDSCs was correlated with CLL tumor progression and a poor prognosis for CLL patients, and CD14^+^HLA-DR^low/−^ MDSCs were significantly correlated with the presence of CD4^+^ T and CD5^+^CD19^+^ cells in CLL patients, which could significantly inhibit the CD4^+^ T-cell immune response, contributing to CLL cell progression in CLL patients.

## Introduction

Significant progress has been made in the development of prognostic factors for chronic lymphocytic leukemia (CLL), which is difficult to predict using clinical parameters, as some patients exhibit indolent disease for decades while others suffer from rapid progression of the disease and require early treatment (1). Furthermore, the development of CLL is associated with immune suppression in the host, which contributes to the failure to mount an effective immune response against the cancer cells (2); however, no prognostic factors for indicating the host immune status of CLL patients exist at present.

Myeloid-derived suppressor cells (MDSCs), a subset of T cells, are a heterogeneous population of immature myeloid cells with immunosuppressive function. Therefore, the presence of MDSCs in the tumor microenvironment has been widely investigated (3). Previously, a novel population of MDSCs, termed cluster of differentiation 14 (CD14)^+^ human leukocyte antigen (HLA)-DR^low/−^ MDSCs, were identified in melanoma, hepatocarcinoma and B-cell non-Hodgkin lymphoma patients (4–6), indicating that low or lacking HLA-DR expression is associated with a CD14^+^ cell subset, which is highly suppressive of lymphocyte function.

Therefore, the present study analyzed CD14^+^HLA-DR^low/−^ MDSCs from 49 CLL patients, monoclonal B-cell lymphocytosis (MBL) patients and healthy volunteers. Furthermore, the correlation between CLL patient survival and CD14^+^HLA-DR^low/−^ expression was also investigated.

## Patients and methods

### Patients

Peripheral blood samples were collected from 49 untreated CLL patients (males, 34; females, 15; mean age, 63 years; age range, 50–85 years) and 23 MBL patients (males, 18; females, 5; mean age, 67 years; age range, 54–86 years) who met the World Health Organization diagnostic criteria for CLL and MBL (7), as well as 21 age-matched healthy controls (males, 15; females, 6; mean age, 57 years; age range, 48–65 years). The MBL patients were screened from a cohort of healthy adults from the general population with normal peripheral blood lymphocyte counts using the sensitive technique of multicolor flow cytometry. Individuals with abnormal peripheral blood lymphocyte counts were denoted as MBL cases and the remainder were classified as controls. Any individuals with immune or chronic infectious diseases were excluded from the present study. Of the 49 CLL patients, 12, 18, 13 and six were classified as stages I, II, III and IV, respectively, based on the Rai staging criteria (8). All patients provided informed consent in accordance with the Declaration of Helsinki and the study was approved by the Zhejiang Province People’s Hospital Review Board (Hanzhou, China).

### Flow cytometry

For flow cytometry diagnosis and prognosis of the CLL patients, the following mouse anti-human monoclonal antibodies (MoAbs), labeled with fluorescein isothiocyanate (FITC), phycoerythrin (PE), allophycocyanin (APC) and peridinin chlorophyll-a protein (PerCP) were used: CD5 (cat. no. A07710), CD10 (cat. no. A07708), CD19 (cat. no. A07708), CD20 (cat. no. A07708), CD22 (cat. no. IM0779u), CD23 (cat. no. A07710), CD38 (cat. no. A07778), CD79a (cat. no. A07705), Flinders Medical Centre (FMC-7; cat. no. A07791), κ (cat. no. A07706), λ (cat. no. A07706) and ζ-chain-associated protein kinase-70 (ZAP-70; cat. no. 731902). For the lymphocyte subset analyzed, the following mouse anti-human MoAbs labeled with FITC, PE, APC and PerCP were used: CD3 (cat. no. 6607013), CD4 (cat. no. A07751), CD8 (cat. no. 6607013), CD14 (cat. no. 6603262), CD16 (cat. no. 6607073), CD19 (cat. no. 6607073), CD25 (cat. no. IM0478u), CD56 (cat. no. 6607073), CD127 (cat. no. IM1980u) and HLA-DR (cat. no. IM0463u). All of the abovementioned products were purchased from Immunotech (Marseille, France). Cells stained with separate antibodies were defined as CD3^+^ T cells (CD3^+^), CD4^+^ T cells (CD3^+^CD4^+^), CD8^+^ T cells (CD3^+^CD8^+^), regulatory T cells (T_reg_ cells; CD4^+^CD25^high^CD127^low^ T helper), B cells (CD3^−^CD19^+^), natural killer (NK; CD3^−^CD16^+^CD56^+^), MDSCs (CD14^+^HLA-DR^low/−^) and CD5^+^CD19^+^ cells. CD38 and cytoplasmic ZAP-70 expression were analyzed within the CD19^+^CD5^+^ lymphocytes. Additionally, the expression the signal transduction molecule ZAP-70 was provided as the percentage of mean fluorescence intensity (MFI) of the gated events, and regarded as high when >20%. The expression of CD38 was also provided as a percentage of the MFI of the gated events, and was regarded as high when >30%.

### Immunoglobulin heavy chain (IGHV) gene mutation

The IGHV gene mutation status of the CD19^+^CD5^+^ cells of all patients was assessed as previously described (9). Briefly, IgVH gene mutations were detected by polymerase chain reaction and specific primers (cat. no. 5-101-0010), using the IGH Somatic Hypermutation Assay for Gel Detection kit (Invivoscribe Technologies, Inc., San Diego, CA, USA), according to the manufacturer’s instructions. Sequences exhibiting <98% homology with the corresponding germline IGHV genes were considered to be mutated.

### Statistical analysis

Statistical analysis was performed using GraphPad Prism software (version 5.01; GraphPad Software, Inc., La Jolla, CA, USA) and the following statistical tests were performed: The log-rank test, the Mann Whitney U test and Spearman’s rank correlation. In addition, quantitative data are presented as the mean ± standard deviation. ^*^P<0.05, ^**^P<0.01 and ^***^P<0.001 were considered to indicate a statistically significant difference.

## Results

### Correlation between CD14^+^HLA-DR^low/−^ MDSC upregulation and CLL tumor progression

**Figure 1:**
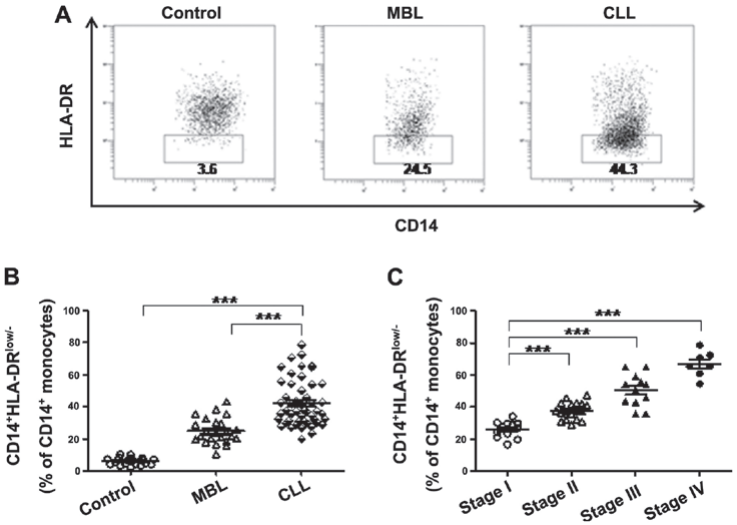
The CD14^+^HLA-DR^low/−^ MDSCs are upregulated in CLL patients. (A) Representative HLA-DR expression in CD14^+^ monocyte population of the control group, MBL patients and CLL patients. (B) Frequency of CD14^+^HLA-DR^low/−^ MDSCs in CD14^+^ monocytes of CLL patients (42.1±14.5%; n=49) were significantly elevated compared with MBL (24.95±8.1%; n=23) and control (6.1±2.2%; n=21) patients (both were P<0.0001). (C) Frequency of CD14^+^HLA-DR^low/−^ MDSCs in CD14^+^ monocytes were significantly increased in advanced stages compared with the stage I CLL patients (stage II: n=18; P<0.0001; stage III: n=12; P<0.0001; stage IV: n=7; P=0.0005). ^***^P<0.001. CD14, cluster of differentiation 14; HLA, human leukocyte antigen; MDSCs, myeloid-derived suppressor cells; CLL, chronic lymphocytic leukemia; MBL, monoclonal B cell lymphocytosis.

Analysis of the frequency of CD14^+^HLA-DR^low/−^ MDSC cells in 49 CLL, 23 MBL and 21 control cases revealed that the frequency of CD14^+^HLA-DR^low/−^ cells was significantly elevated in CLL patients compared with MBL and control cases (Fig. 1A and B), where MBL is a condition that resembles CLL but does not meet all of the criteria for CLL (10). The clinicopathological data of the studied patients and control cases, including the number of subjects, median age, gender, Rai stage, IGHV mutation status, CD38 status and ZAP-70 status, were recorded (Table I). Furthermore, statistical analyses revealed that CD14^+^HLA-DR^low/−^ cells were significantly associated with the clinical stage of disease in CLL patients (Fig. 1C), indicating that these cells may participate in the progression of CLL.

### CD14^+^HLA-DR^low/−^ MDSC and poor prognosis for CLL patients

**Figure 2:**
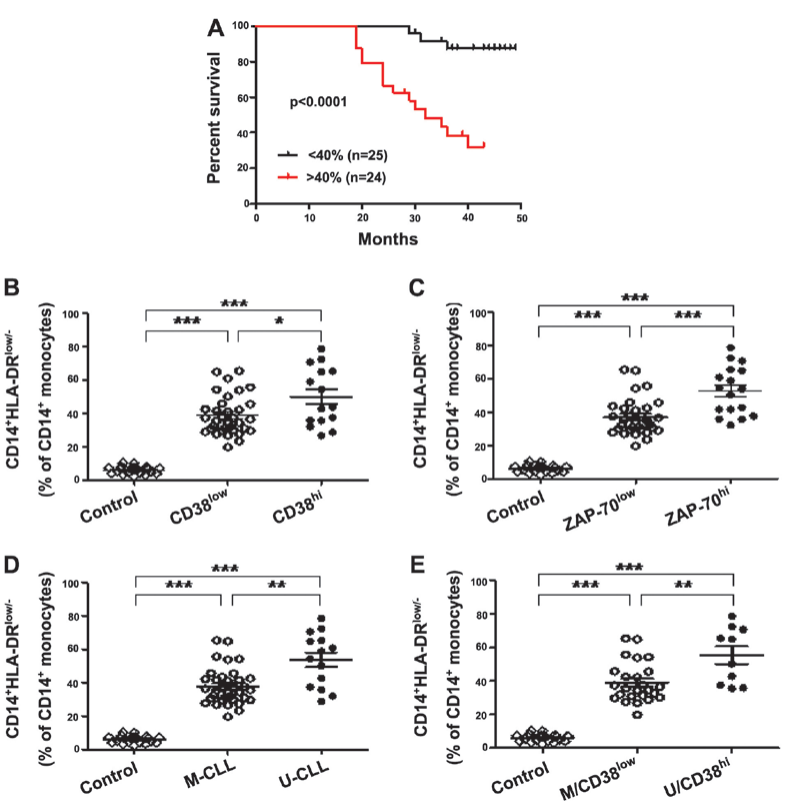
Upregulation of CD14^+^HLA-DR^low/−^ MDSCs predicts poor survival in CLL patients. (A) Kaplan-Meier curves of CLL patients with <40% (n=25) versus >40% (n=24) CD14^+^HLA-DR^low/−^ MDSCs in the CD14^+^ monocytes (log-rank test, P<0.0001). (B) Frequency of CD14^+^HLA-DR^low/−^ MDSCs in CD14^+^ monocytes of CLL patients stratified by CD38 expression levels compared with the control. A significantly higher frequency of CD14^+^HLA-DR^low/−^ MDSCs were identified in CD38^high^ (n=15) and CD38^low^ patients (n=34) compared with the healthy controls (n=21) (both P<0.0001). The CD38^high^ subgroup tended to have higher frequency of CD14^+^HLA-DR^low/−^ MDSCs compared with the CD38^low^ subgroup (P=0.0335). (C) A significantly higher frequency of CD14^+^HLA-DR^low/−^ MDSCs in CD14^+^ monocytes was identified in ZAP-70^high^ (n=17) and ZAP-70^low^ patients (n=32) compared with the healthy controls (n=21) (both P<0.0001), and the ZAP-70^high^ subgroup exhibited a higher frequency than the ZAP-70^low^ subgroup (P=0.0003). (D) A significantly higher frequency of CD14^+^HLA-DR^low/−^ MDSCs in CD14^+^ monocytes was identified in U-IGHV (n=14) and M-IGHV patients (n=35) compared with in the healthy controls (n=21) (both P<0.0001), and the ZAP-70^high^ subgroup exhibited a higher frequency than the ZAP-70^low^ subgroup (P=0.0019). (E) A significantly higher frequency of CD14^+^HLA-DR^low/−^ MDSCs in CD14^+^ monocytes was identified in U/CD38^high^ (n=10) and M/CD38^low^ patients (n=35) compared with in the healthy controls (n=21) (both P<0.0001), and the U/CD38^high^ subgroup exhibited a higher frequency than the M/CD38^low^ subgroup (P=0.0052). ^*^P<0.05, ^**^P<0.01 and ^***^P<0.001. CD14, cluster of differentiation 14; HLA, human leukocyte antigen; MDSCs, myeloid-derived suppressor cells; ZAP-70, ζ-chain-associated protein kinase-70; CLL, chronic lymphocytic leukemia; M, mutated; U, unmutated.

Most notably, the present study identified that the overall survival time in CLL patients whose samples were obtained prior to the administration of any therapy (n=49) was significantly different between those exhibiting low and high CD14^+^HLA-DR^low/−^ expression levels, and that CD14^+^HLA-DR^low/−^ expression was inversely correlated with survival time (P<0.0001; Fig. 2A). Furthermore, 15 patients in the high CD14^+^HLA-DR^low/−^ expression group (n=24; >40% CD14^+^ monocytes) and three patients in the low expression group (n=25; <40% CD14^+^ monocytes) succumbed to CLL within four years, indicating that CD14^+^HLA-DR^low/−^ MDSCs may be a prognostic factor in CLL patients. CD38, ZAP-70 and the IGHV gene are typical immunophenotypic and genetic prognostic markers (11,12), which have previously been used to predict the survival of CLL patients in the clinic. Firstly, when comparing CLL patients grouped according to CD38 expression, CD14^+^HLA-DR^low/−^ MDSC levels in CD38^low^ and CD38^high^ CLL patients were significantly higher compared with the control group (both P<0.0001). In addition, the CD14^+^HLA-DR^low/−^ MDSC count was significantly higher in the CD38^high^ subgroup compared with the CD38^low^ subgroup (P=0.0335; Fig. 2B), the CD14^+^HLA-DR^low/−^ MDSC frequency was significantly elevated in the ZAP-70^low^ and ZAP-70^high^ CLL patients when compared with the control group (both P<0.0001), and was significantly higher in the ZAP-70^high^ subgroup compared with ZAP-70^low^ subgroup (P=0.0003) (Fig. 2C). Following data analysis based on the mutational status of IGHV, a similar frequency pattern was observed: The frequency of CD14^+^HLA-DR^low/−^ MDSCs was significantly higher in the IGHV-unmutated (U-CLL) and IGHV-mutated (M-CLL) CLL patients when compared with the control (both P<0.0001), and these MDSCs were significantly more elevated in the U-CLL patients compared with the M-CLL patients (P=0.0019) (Fig. 2D). Furthermore, when patients were divided based on combinations of good (M-CLL/CD38^low^) or poor (U-CLL/CD38^high^) prognosis markers, the M-CLL/CD38^low^ and U-CLL/CD38^high^ subgroups of patients exhibited significantly higher CD14^+^HLA-DR^low/−^ MDSC frequency compared with the controls (both P<0.0001), and the CD14^+^HLA-DR^low/−^ frequency in the U-CLL/CD38^high^ subgroup was higher than in the M-CLL/CD38^low^ subgroup (P=0.0052) (Fig. 2E). In the present study, it was identified that CD14^+^HLA-DR^low/−^ MDSCs are associated with CD38 and ZAP-70 expression, as well as IGHV mutations. Thus, CD14^+^HLA-DR^low/−^ MDSCs may be prognostic factors in CLL patients.

### CD14^+^HLA-DR^low/−^ MDSCs predominantly inhibit the CD4^+^ T-cell response during CLL disease progression

**Figure 3:**
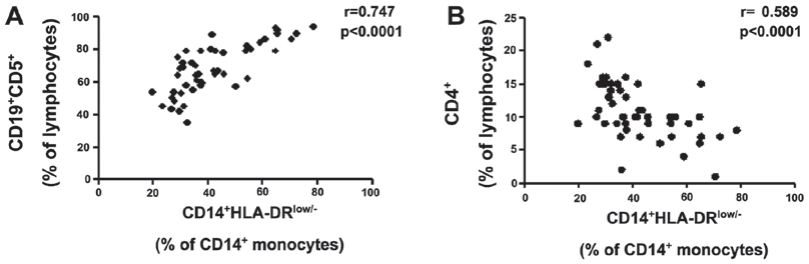
The frequency of the CD14^+^HLA-DR^low/−^ myeloid-derived suppressor cells (MDSCs) was significantly correlated with the frequency of CD4^+^ T and CD5^+^CD19^+^ cells in chronic lymphocytic leukemia patients. The frequency of CD14^+^HLA-DR^low/−^ MDSCs were (A) positively correlated with the frequency of CD5^+^CD19^+^ B cells (r=0.747; P<0.0001; n=49) and (B) negatively correlated with the frequency of CD4^+^ T cells (r=0.589; P<0.0001; n=49). CD14^+^HLA-DR^low/−^, cluster of differentiation 14^+^ human leukocyte antigen^−^DR^low/−^.

As previously established, MDSCs suppress immunity by disrupting the innate and adaptive immune responses (3). In the present study, the frequency of various lymphocyte subsets were assessed, and it was identified that the frequency of CD3^+^ T, CD4^+^ T, CD8^+^ T and NK cells in CLL patients were significantly reduced compared with in the MBL patients and the control group (Table II). By contrast, B, T_reg_ and CD19^+^CD5^+^ cells were significantly elevated in CLL patients compared with MBL patients and the control group (Table II). Furthermore, Spearman’s correlation analysis revealed that CD14^+^HLA-DR^low/−^ MDSCs were significantly positively correlated with CD19^+^CD5^+^ cells (Fig. 3A) and negatively correlated with the CD4^+^ T cells (Fig. 3B); however, no significant correlation was identified with CD3^+^ T, B, T_reg_ or NK cells (data not shown). Data obtained in the present study was inconsistent with previous reports, which described MDSCs as potent inducers of T_reg_ cells in liver cancer patients (4). However, in general, CD14^+^HLA-DR^low/−^ MDSCs appear to disturb systemic immunity during CLL disease progression, predominantly by inhibiting the CD4^+^ T-cell response. Additionally, no correlation was identified between CD14^+^HLA-DR^low/−^ MDSCs, and age, serum hemoglobin concentration, platelet and white blood cell count, Rai stage and lymphocytosis (data not shown).

## Discussion

Currently, the use of clinical, biological and genetic parameters allows CLL patients to be diagnosed and characterized with a mild, intermediate or aggressive onset and prognosis of this heterogeneous disease (13). However, excepting allogeneic stem cell transplantation, there is no curative treatment available for CLL patients, possibly due to CLL patients exhibiting a defective immune antitumor response. Furthermore, in individuals with established CLL, MDSCs are likely to be a major factor in reducing the efficacy of therapeutic treatment strategies, which require an immunocompetent host (14). Therefore, improved understanding of the immune status of individual CLL patients may be useful for determining which patients may exhibit indolent disease for a number of decades, and which patients may progress rapidly and require early treatment. In addition, the elimination of MDSCs is a priority for CLL patients who are candidates for active immunotherapy.

Presently, there is a requirement to monitor the number of immunosuppressive CD14^+^HLA-DR^low/−^ MDSCs (>40% CD14^+^ monocytes) in CLL patients, as the survival time from diagnosis is currently <3 years. However, additional multivariate statistical analysis with other prognostic factors in long-term multicenter studies should initially be performed to clarify the observations of the current study, as the follow-up period of CLL patients in the present study was only conducted for ~4 years. Furthermore, the specific immunosuppressive function of CD14^+^HLA-DR^low/−^ MDSCs in CLL patients requires further investigation.

In conclusion, to the best of our knowledge, the present study reports for the first time a population of immunosuppressive monocytes, characterized by the CD14^+^HLA-DR^low/−^ phenotype, which were significantly elevated in CLL patients and were poor predictors of survival in CLL patients (n=49). In addition, these CD14^+^HLA-DR^low/−^ MDSCs were associated with the prognostic factors CD38, ZAP-70 and IGHV. Finally, Spearman’s correlation analysis revealed that the poor survival of upregulated CD14^+^HLA-DR^low/−^MDSC CLL patients predominantly occurred via the inhibition of the CD4^+^ T-cell response, resulting in a dysregulation of the balance of T-cell subsets *in vivo*, including significant proliferation of CD5^+^CD19^+^ cells in CLL patients. Thus, CD14^+^HLA-DR^low/−^ MDSCs may be used as a novel prognostic marker for the survival of CLL patients and eliminating CD14^+^HLA-DR^low/−^ MDSCs may represent a promising strategy for the treatment of CLL patients.

## Figures and Tables

**Table I:** Clinicopathologic characteristics of the different groups of patients.

	Chronic lymphocytic lymphocytosis	Monoclonal B cell leukemia	Control
Subjects, n	49	23	21
Median age (range), years	63 (50–85)	67 (54–86)	57 (48–65)
Gender, n			
Male	34	18	15
Female	15	5	6
Rai stage, n			
0 – II	30	NA	NA
III – IV	19	NA	NA
Immunoglobulin heavy chain mutation status, n			
Mutated (≥2%)	31	NA	NA
Unmutated (<2%)	18	NA	NA
Cluster of differentiation 38 status, n			
Low (<30%)	32	NA	NA
High (≥30%)	17	NA	NA
ζ-chain-associated protein kinase-70 status, n			
Low (<20%)	30	NA	NA
High (≥20%)	19	NA	NA

NA, not applicable.

**Table II:** Lymphocyte subsets in the CLL, MBL and control groups.

	Mean ± standard deviation, %		
	
Lymphocyte	CLL	MBL	Control
CD3^+^	14.61±3.72	35.68±9.35	69.42±7.39
CD4^+^	8.15±6.38	22.73±5.15	35.17±4.56
CD8^+^	6.57±2.62	21.61±7.33	23.46±4.78
CD3^−^CD16^+^CD56^+^NK	3.92±2.40	6.85±1.77	8.63±2.77
CD4^+^CD25^+/high^CD127^low/−^	10.09±3.21	5.84±1.76	4.38±1.42
CD3^−^CD19^+^	68.74±14.56	39.46±5.60	12.31±4.29
CD5^+^CD19^+^	51.68±9.43	35.16±6.18	2.85±1.36

CLL, chronic lymphocytic leukemia; MBL, monoclonal B cell lymphocytosis; CD cluster of differentiation; NK, natural killer.
